# Terminology inaccuracies in the interpretation of imaging results in detection of cervical lymph node metastases in papillary thyroid cancer

**DOI:** 10.1530/EC-12-0050

**Published:** 2012-10-10

**Authors:** Mubashir Mulla, Klaus-Martin Schulte

**Affiliations:** 1 Department of Endocrine Surgery King's College Hospital Denmark Hill, London, SE5 9RS UK; 2 King's College London London UK; 3 King's Health Partners London UK

**Keywords:** papillary thyroid cancer, cervical lymph node, metastases, ultrasound, CT, sensitivity, negative predictive value, MRI

## Abstract

Cervical lymph nodes (CLNs) are the most common site of metastases in papillary thyroid cancer (PTC). Ultrasound scan (US) is the most commonly used imaging modality in the evaluation of CLNs in PTC. Computerised tomography (CT) and ^18^fluorodeoxyglucose positron emission tomography (^18^FDG PET–CT) are used less commonly. It is widely believed that the above imaging techniques should guide the surgical approach to the patient with PTC.

**Methods:**

We performed a systematic review of imaging studies from the literature assessing the usefulness for the detection of metastatic CLNs in PTC. We evaluated the author's interpretation of their numeric findings specifically with regard to ‘sensitivity’ and ‘negative predictive value’ (NPV) by comparing their use against standard definitions of these terms in probabilistic statistics.

**Results:**

A total of 16 studies used probabilistic terms to describe the value of US for the detection of LN metastases. Only 6 (37.5%) calculated sensitivity and NPV correctly. For CT, out of the eight studies, only 1 (12.5%) used correct terms to describe analytical results. One study looked at magnetic resonance imaging, while three assessed ^18^FDG PET–CT, none of which provided correct calculations for sensitivity and NPV.

**Conclusion:**

Imaging provides high specificity for the detection of cervical metastases of PTC. However, sensitivity and NPV are low. The majority of studies reporting on a high sensitivity have not used key terms according to standard definitions of probabilistic statistics. Against common opinion, there is no current evidence that failure to find LN metastases on ultrasound or cross-sectional imaging can be used to guide surgical decision making.

## Introduction

Papillary thyroid cancer (PTC) is the most common thyroid cancer. Metastases from PTC most commonly involve the cervical lymph nodes (CLNs). The incidence of CLN metastases (CLNM) is reported between 30 and 80% (1, 2). Some studies have concluded that in PTC with no other adverse features, the state of the CLNs does not influence prognosis (3). Despite the high incidence of metastases, prophylactic CLN dissection has been discouraged because CLNM has not been considered to be a prognostic factor for survival (4, 5). However, the presence of CLNM has been shown to increase local recurrence rates (6, 7) to up to 31% of patients (8). There is an ongoing debate about the role of systematic central lymph node (LN) dissection in PTC (9).

The preoperative diagnosis of LN metastasis is important for selecting surgical strategies (10). Ultrasound scan (US) is currently the most favoured diagnostic modality to evaluate nodal status preoperatively. It is recommended for this purpose by the American Thyroid Association (ATA) (11) and supported by other thyroid associations (12). Confirmation of metastatic LN with suspicious features on US is achieved by US-guided fine needle aspiration for cytology and/or measurement of thyroglobulin in needle washout (11). Although this method is useful, it does improve sensitivity of US. A number of studies have reported the ‘usefulness’ and ‘diagnostic accuracy’ of US and other imaging modalities, namely computerised tomography (CT), magnetic resonance imaging (MRI) and ^18^fluorodeoxyglucose positron emission tomography (^18^FDG PET–CT) in the evaluation of metastatic cervical LN in PTC.

We set out to understand whether the commonly reported ‘high sensitivity’ of imaging techniques to exclude LN metastasis is based on factual evidence. For this purpose, we have tabled the actual data reported by the authors and have analysed whether their use of the terms ‘sensitivity’ and ‘negative predictive value’ (NPV) conforms to textbook definitions of probabilistic statistics. This study does not set out to analyse or critically comment on the impact of LN metastases on prognosis. Our analysis does not directly impact decision making as to the choice of surgical intervention.

## Materials and methods

In designing this systematic review, we reviewed published work available from the NIH database PubMed (http://www.ncbi.nlm.nih.gov/pubmed) and Thompson resource ‘ISI Web of Knowledge’ (http://apps.isiknowledge.com/). Accordingly, we included the following criteria for studies to be entered into the systematic review.

- Studies involving imaging modalities in detection of CLNs in PTC published in the last 17 years.
- All studies had to be published in English.

The authors searched for articles reported over the last 17 years from 1995 up to June 2011 in PubMed, with the combination of search terms ‘papillary thyroid cancer’, ‘LNs dissection’, ‘sensitivity’, specificity, ‘therapeutic LN dissection, US, CT, MRI’ and ‘^18^FDG PET–CT’. The search was restricted to the presence of one or more of these key words in the title or abstract of the articles.

The preliminary search using these terms yielded 1129 publications. The abstracts of these were read and 32 publications with potential data identified. These 32 publications were read in full text and scrutinised for the presence of relevant data. This process identified 28 studies appropriate for analysis based on the criteria set out above.

Sixteen studies looked at sensitivity of US (13, 14, 15, 16, 17, 18, 19, 20, 21, 22, 23, 24, 25, 26, 27, 28). Eight studies provided data for CT (13, 15, 21, 24, 25, 27, 29, 30), one study involved MRI (31) and three studies provided data for ^18^FDG PET–CT (21, 30, 32).

## Results

### US

Seven studies were identified assessing the use of US in detecting central LN in PTC (Table 1). Out of these, only four studies calculated the sensitivity and NPV correctly. Out of the 11 studies providing data for lateral LN detection, only one (19) was accurate in their calculation of sensitivity and NPV. Five studies provided clustered results for both central and lateral CLN detection, out of which only one (16) calculated the sensitivity and NPV correctly.

### Computerised tomography

A total eight studies were included. Five provided data for central CLN out of which in only one (27), the calculations were correct (Table 2). For lateral LN, seven studies provided the data; however, none of these calculated sensitivity and NPV correctly. One study (30) with clustered results for both central and lateral CLN again was incorrect in the calculations.

### Magnetic resonance imaging

One study provided data for use of MRI in detection of cervical LN, which was again not calculated in a correct manner (Table 3).

### ^18^Fluorodeoxyglucose positron emission tomography–computerised tomography

Three studies provided information for this, one for lateral and two with combined results. None of these studies calculated the sensitivity accurately (Table 3).

In summary, sensitivity was calculated incorrectly in 15 studies (13, 14, 15, 17, 18, 21, 22, 23, 24, 25, 27, 29, 30, 31, 32) and the NPV was calculated incorrectly in 13 studies (13, 14, 15, 17, 21, 23, 24, 25, 27, 29, 30, 31, 32). The most common error made in the calculation of sensitivity and NPV was to assume the number of false negative (FN) LNs to be zero.

## Discussion

The frequency of metastases to the cervical LN in PTC is in the region 60–70% (9, 33). The presence of LN metastases is known to be associated with regional recurrence (34). Micro-metastases (<2 mm in diameter) have an incidence of up to 90% depending on the technique used. The clinical implications of this are unclear and possibly less significant than macro-metastases (11). For planning any cancer treatment, it is imperative to have accurate staging as it impacts on the treatment strategy, prognosis and ultimately on long-term survival (35). In PTC, the importance of a thorough preoperative evaluation and subsequent appropriate initial surgery has been emphasised in many studies (7, 36).

US is the most popular imaging modality used in preoperative evaluation of cervical LN in PTC and is recommended by ATA guidelines (11). CT and MR are other modalities utilised. Preoperative staging in PTC therefore largely depends on these imaging modalities. The assessment of ‘usefulness’ and ‘diagnostic accuracy’ of these imaging modalities relies on four criteria, namely sensitivity, specificity, positive predictive value (PPV) and NPV. The ideal scenario for any imaging modality used for preoperative staging of cancer would be a high score on all the aforementioned values. The most serious error in cancer surgery would be under treatment, and this error would be provoked by under-staging or in other words by a false high sensitivity or NPV of the test(s) employed. This argument is not faulted by any claim that the presence of the finding (metastatic LN) tested for is irrelevant. This holds true for logical reasons, as this reasoning would conform with the fallacy of the appeal to consequences or ‘argumentum ad consequentiam’. In addition, it has been shown that CLN status at presentation affects long-term outcomes (37). In the absence of exact data how LN metastases impact outcome in PTC, a merely hypothetical and not factual statement does not substitute for a rational debate around methodological issues of identifying LN metastases.

Sensitivity is defined as the ‘proportion of true positives (TP) that are correctly identified by the test’ (38). In other words, it is the ability of a test to identify those patients with the disease (39). It is defined by the formula TP/TP+FN.

‘NPV’ is defined as the ‘proportion of subjects with a negative test result who are correctly diagnosed’ (38). In other words, it is the likelihood of a patient not having the disease when the test result is negative (39). It is defined by the formula TN/TN+FN (true negatives (TN)).

It is obvious from the above formulae that for calculation of sensitivity and NPV, FN values are required. Although specificity and PPV are important, it is the sensitivity and NPV that define a test in cancer staging as these represent those cases ‘missed’ by the test.

The only empiric method to obtain FN values in PTC is dissection of all cervical LN during surgery irrespective of image findings followed by comparison of histology results to those detected by preoperative imaging. This alone will ascertain that all ‘FN’ cases are included.

This systematic review provides evidence that out of the 16 studies providing values for sensitivity and NPV of US in cervical LN detection, only six studies calculated these values accurately leaving ten studies with either incomplete or inaccurate data (Tables 4 and 5). For studies involving CT, out of eight studies included, only one (27) provided accurate results for sensitivity and NPV. The results from the remaining seven studies are incorrect (Tables 4 and 5). The same applies to ^18^FDG PET–CT and MR studies; all four studies included calculated the values incorrectly. Overall, only seven out of 28 studies included have performed the calculations correctly, which in effect means that the results and conclusions from the 21 studies are incorrect.

These inaccuracies reflect misunderstandings or simply incorrect definitions of the terms ‘sensitivity’ and ‘NPV’ in the context of imaging in PTC. In all those studies that have calculated sensitivity and NPV incorrectly, all cervical LN were not dissected, hence providing unreliable ‘FN’ values.

A recent meta-analysis by Wu *et al*. (40) looked at the accuracy of US in the detection of metastatic LN. They provide ‘pooled sensitivity’ figures of 0.72 and 0.63 for patient- and region-based LND respectively. This pooled sensitivity is essentially weighted sensitivity calculated from studies some of which have performed therapeutic LND. The results provided by the meta-analysis are incorrect as FN values would not have been known. It seems to us that sensitivity is a widely misunderstood term certainly in the case of imaging in PTC.

The foundation of scientific analysis is correct methodology. Based on this approach, the mean sensitivity and NPV of US was 36.2 and 57.3% respectively. The only study, which provided correct results for CT, had a sensitivity of 67% and NPV of 80% for central compartment only (27).

From the available data, US or indeed CT cannot be considered reliable imaging modalities for cervical LN detection in PTC. It needs to be acknowledged that US is operator dependent and individual results may be better than what has been published in the peer-reviewed literature. However, such operator dependency puts obvious limitations to the use of the results obtained and requires rigorous internal audit procedures. Properly designed studies calculating FN correctly and thereby providing accurate values for sensitivity and NPV are required to resolve this issue.

It is beyond the remit of this study to provide recommendations on the surgical approach to potential lateral LN metastases. Our purpose was to look at the quality of the evidence underlying eventual current assumptions and recommendations.

## Conclusion

The majority of data on sensitivity and accuracy of imaging for preoperative detection of LN in PTC is misleading and hence cannot be relied upon for its preoperative staging. The few studies reporting accurate figures have identified low sensitivities to detect locoregional LN metastases of PTC. Reliance on preoperative imaging for planning of the surgical approach is currently unsupported by published evidence. Studies with appropriate design are needed to inform the discussion.

## Figures and Tables

**Table 1:** Ultrasonography in detection of cervical LN in PTC. Figures in brackets indicate erroneous calculations.

**Study**	**Total patients** (*n*)	**Sensitivity** (%)	**Specificity** (%)	**PPV** (%)	**NPV** (%)
Central					
(19)	600	10.5	99.1	90	59.5
(26)	231	29	91	82	47
(28)	133	61	93	92	63
(25)	165	(38)	93	77	(70)
(24)	37	(55)	69	77	(44)
(27)	299	53	80	61	74
(13)	589	(47.2)	94.8	90.4	(63.5)
Lateral					
(19)	560	27.2	96.5	93.3	43
(21)	26	(41.3)	97.4	73.1	(90.6)
(22)	551	(83.5)	97.7	88.8	NA
(26)	361	NA	NA	98	NA
(28)	133	NA	NA	NA	NA
(25)	165	(64)	92	83	(82)
(24)	37	(65)	82	86	(59)
(27)	299	(93.9)	25	93.9	(25)
(13)	589	(69.1)	94.8	57.6	(96.8)
(14) a	70	(78.3)	98.7	99.5	(58.2)
(15)	113	(74.3)	82.7	85.3	(70.5)
Combined central and lateral					
(16)	77	36.7	89.3	NA	NA
(17)	63	(70)	100	100	(87.6)
(18)	456	(100)	100	NA	NA
(22) b	219	(90.4)	78.9	93.9	NA
(23)	60	(92)	98	92	(98)
Mean (of correct calculations)		36.2			57.3

a Unclear if all LN were dissected.

b Re-operated group of patients.

**Table 2:** CT in the detection of cervical LN metastases in PTC. Figures in brackets indicate erroneous calculations.

**Study**	**Patients total** (*n*)	**Sensitivity** (%)	**Specificity** (%)	**PPV** (%)	**NPV** (%)
Central LN					
(25)	165	(50)	91	79	(74)
(29)	102	(58)	72	72	(58)
(24)	37	(74)	44	72	(47)
(27)	299	67	79	65	80
(13)	589	(41.9)	97.4	93.6	(65.1)
Lateral LN					
(21)	26	(42.3)	96.6	57.9	(93.8)
(25)	165	(74)	95	89	(86)
(29)	102	(50)	71	50	(63)
(24)	37	(78)	78	86	(68)
(27)	299	(81.7)	100	100	(30.8)
(13)	589	(78.2)	93.3	54.4	(97.7)
(15)	113	(68.6)	78.8	81.4	(65.1)
Combined central and lateral LN					
(30)	11	(75)	87.5	95.9	(50)
Mean (of correct calculations)		67			80

**Table 3:** ^18^FDG PET–CT and MRI in the detection of CLN in PTC. Figures in brackets indicate erroneous calculations.

**Study**	**Total patients** (*n*)	**Sensitivity** (%)	**Specificity** (%)	**PPV** (%)	**NPV** (%)
18-F PET–CT					
Lateral					
(21)	27	(50)	97	65	(94.6)
Combined central and lateral					
(32) a	22	(80)	83	90	(69)
(30) a	11	(35.7)	87.5	90.9	(28)
MRI					
(31)	105	(95)	51	84	(78)

a Patients with local recurrence in cervical LN.

**Table 4:** Study details and validity: central and combined cervical LN.

**Study**	**Patients included**	**Study hypothesis**	**TP**	**TN**	**FP**	**FN**	**Sen**	**Spec**	**PPV**	**NPV**	**Validity of conclusion**
Central											
(31)	28	To determine the ability of MRI imaging to detect the presence of metastatic thyroid cancer in cervical LN	NA	NA	NA	NA	–a	–b	–b	–a	Invalid
(20)	600	Investigate the clinical significance of LN in the central compartment	27	339	3	231	–b	–b	–b	–b	Valid
(26)	231	To prospectively analyse the outcomes of selective LND to determine when prophylactic lateral neck dissection is advisable	40	86	9	96	–a	–b	–b	–a	Invalid
(28) c	133	Evaluation of preoperative US in detecting cervical metastases in PTC	NA	NA	NA	NA	–b	–b	–b	–b	Valid
(27)	299	To compare the diagnostic accuracy of US with that of CT in patients with PTC	NA	NA	NA	NA	–b	–b	–b	–b	Valid
(25)	165	Determine the diagnostic accuracies of US, CT and combined US and CT	20	75	6	32	–a	–b	–b	–a	Invalid
(24)	9	Investigate the diagnostic ability of CT and US of cervical LN in thyroid cancer	NA	NA	NA	NA	–a	–b	–b	–a	Invalid
(13)	589	To evaluate the diagnostic accuracy of preoperative US and CT of the neck	NA	NA	NA	NA	–a	–b	–b	–a	Invalid
(29)	102	Sensitivity, specificity. PPV and NPV calculated	NA	NA	NA	NA	–a	–b	–b	–a	Invalid
Central and lateral					
(17)	63	The usefulness of US in diagnosing cervical LNM in PTC was investigated	14	43	0	6	–a	–b	–b	–a	Invalid
(16)	77	To evaluate the usefulness of US for preoperative staging of PTC	NA	NA	NA	NA	–b	–b	NA	NA	Invalid
(18)	456	Determine the sensitivity of neck US to detect LN metastases	38	NA	NA	418	–a	–b	–b	–a	Invalid
(30)	11	If PET–CT is more accurate than CT for detecting metastatic cervical LN in PTC	21	7	1	7	–a	–b	–b	–a	Invalid
(32) c	22	Evaluate whether FDG PET is feasible as a pre-surgical evaluation modality for I-131 scan-negative thyroid carcinoma patients	45	24	5	11	–a	–b	–b	–a	Invalid
(23)	60	To determine the frequency of occult macroscopic metastasis detected by preoperative US	11	47	1	1	–a	–b	–b	–a	Invalid
(22)	551	Preoperative US will increase detection and assessment of the extent of LNM in PTC	NA	NA	NA	NA	–a	–b	–b	–a	Invalid

Sen, sensitivity; Spec, specificity; PPV, positive predictive value; NPV, negative predictive value; TP, true positives; TN, true negatives; FP, false positives; FN, false negatives.

a Incorrectly calculated.

b Correctly calculated.

c Figures available by levels, no overall figures.

**Table 5:** Study details and validity: lateral LN.

**Study**	**Patients included**	**Study hypothesis**	**TP**	**TN**	**FP**	**FN**	**Sen**	**Spec**	**PPV**	**NPV**	**Validity of conclusion**
(24)	28	Investigate the diagnostic ability of CT and US of cervical LN in thyroid cancer	NA	NA	NA	NA	–a	–b	–b	–a	Invalid
(25)	165	Determine the diagnostic accuracies of US, CT and combined US and CT	20	75	6	32	–a	–b	–b	–a	Invalid
(19)	560	Investigate the prognostic impact of lateral LNM detected by US	98	194	7	261	–b	–b	–b	–b	Valid
(26)	130	To prospectively analyse the outcomes of selective LND to determine when prophylactic lateral neck dissection in advisable	127	0	3	0	–a	–b	–b	–a	Invalid
(28) c	34	Evaluation of preoperative US in detecting cervical metastases in PTC	NA	NA	NA	NA	NA	NA	NA	NA	Invalid
(21)	26	To compare the diagnostic of ^18^F-FDG PET–CT with US	14	229	5	12	–a	–b	–b	–a	Invalid
(13)	589	To evaluate the diagnostic accuracy of preoperative US and CT of the neck	NA	NA	NA	NA	–a	–b	–b	–a	Invalid
(14)	70	To investigate the usefulness and limits of US in PTC	199	76	1	55	–a	–b	–b	–a	Invalid
(15)	112	To evaluate the most accurate criteria using US and CT in predicting lateral LN in PTC	NA	NA	NA	NA	–a	–b	–b	–a	Invalid
(29)	102	Sensitivity, specificity. PPV and NPV calculated	NA	NA	NA	NA	–a	–b	–b	–a	Invalid
(27)	53	To compare the diagnostic accuracy of US with that of CT in patients with PTC	NA	NA	NA	NA	–a	–b	–b	–a	Invalid

Sen, sensitivity; Spec, specificity; PPV, positive predictive value; NPV, negative predictive value; TP, true positives; TN, true negatives; FP, false positives; FN, false negatives.

a Incorrectly calculated.

b Correctly calculated.

c Figures available by levels, no overall figures.
